# Simplified ABO-OGS orientation improves training of orthodontic bracket positioning for undergraduate dental students

**DOI:** 10.1186/s12909-025-06839-y

**Published:** 2025-02-20

**Authors:** Jiali Liu, Yuchun Zou, Jing Huang, Ziqin Chen, Jia Hu, Linyu Xu, Jiehua Su

**Affiliations:** 1 School and Hospital of Stomatology, Fujian Medical University, Fuzhou City, Fujian Province P. R. China; 2 Orthodontics Department, School and Hospital of Stomatology, Fujian Medical University, Yangqiao Zhong Road No 246, Fuzhou City, Fujian Province P. R. China; 3 Fujian Key Laboratory of Oral Diseases, School and Hospital of. Stomatology, Fujian Medical University, Jiaotong Road No 88, Fuzhou City, Fujian Province P. R. China

**Keywords:** ABO-OGS, Goal-oriented learning, Bracket bonding course, Simplified ABO-OGS-oriented training, Hybrid teaching methods, Dentistry, Orthodontics

## Abstract

**Background:**

Mastery of orthodontic bracket bonding is an essential component of the undergraduate orthodontic curriculum. Traditional bracket bonding training using simple plaster models has some shortcomings, including a disconnect from clinical practice and poor perception of the criteria and accuracy. This study aims to optimize the bracket bonding course by comparing the traditional teaching method with simplified American Board of Orthodontics Objective Grading System (ABO-OGS)-oriented training methods.

**Methods:**

Fourth-year dental students from Fujian Medical University, spanning the 2015 to 2017 cohorts, participated in the orthodontic bracket bonding course. Students from these three cohorts were trained using the traditional plaster model method, the digital 2D ABO-OGS-oriented method, and the typodont ABO-OGS-oriented method, respectively. After the course, students and teachers completed a six-level Likert scale questionnaire to evaluate the teaching objectives, methods, and efficacy.

**Results:**

Both teachers and students agreed that the traditional bracket bonding training and the simplified ABO-OGS-oriented bracket bonding training were effective, with most students mastering the bracket bonding procedure. The simplified ABO-OGS-oriented bracket bonding was perceived as more novel and engaging compared to the traditional method (*P* < 0.05). However, the digital 2D ABO-OGS method was significantly less effective than the other two methods (*P* < 0.05). Instructors rated the typodont ABO-OGS-oriented training higher than the students did (*P* < 0.05).

**Conclusion:**

Both the traditional and simplified ABO-OGS-oriented courses for bracket bonding yielded favorable results, with the simplified ABO-OGS approach being more engaging and innovative. The findings emphasize the critical role of hands-on practice in achieving optimal proficiency in bracket bonding techniques. A hybrid educational model that integrates digital 2D or 3D ABO-OGS-oriented training with offline practical sessions shows considerable potential for qualifying training and examination of orthodontic residency students.

**Supplementary Information:**

The online version contains supplementary material available at 10.1186/s12909-025-06839-y.

## Introduction

Accurate positioning of orthodontic brackets is pivotal in achieving optimal outcomes in orthodontic treatments. Often regarded as the cornerstone of orthodontic treatment, precise bracket bonding is indispensable for successful procedures [1–3]. Mastery of accurate bracket positioning techniques constitutes a fundamental competency for orthodontists [4]. However, undergraduate dental students encounter substantial challenges in acquiring proficiency in orthodontic bracket bonding techniques, particularly within the constraints of limited timeframes, such as a four-credit-hour course. This difficulty is further compounded when training relies on direct bonding using traditional plaster casts, which provide limited opportunities for objective feedback [5].

In conventional orthodontic bracket bonding courses, students are instructed to place brackets at the center of the clinical crown of the tooth [6]. Despite this, they frequently lack a clear and objective understanding of the quality of their bracket positioning. Typically, assessments of bracket positioning rely on subjective evaluations by instructors, who base their judgments on clinical experience. This evaluative method does not provide students with an objective appraisal of their work, leading to inconsistent learning outcomes [7]. Consequently, there is an urgent need to reform teaching methodologies to improve teaching efficacy and elevate educational standards.

The simplified ABO-OGS-oriented approach, grounded in the principles of the American Board of Orthodontics Objective Grading System (ABO-OGS), effectively addresses these challenges by providing a standardized and objective framework for evaluating bracket positioning [8, 9]. This approach facilitates the alignment of instructional objectives with measurable outcomes, thereby bridging the gap between teaching goals and student performance assessment.

Goal-Based Learning (GBL), also referred to as Objective-Based Learning, introduced by American educator and psychologist Benjamin Bloom, has been effectively integrated into diverse educational fields, yielding favorable outcomes [10]. GBL emphasizes student-centered pedagogy by focusing on clearly defined teaching objectives. It is now widely utilized in medical education, demonstrating its effectiveness in various contexts. For instance, it has been shown to significantly enhance the efficiency of undergraduate dental students in learning anatomy, enable medical students to acquire diagnostic radiology skills more effectively and PBL-based dental trauma case enhance predoctoral students’ learning outcomes and confidence in managing simulated scenarios, and so on [7, 11–13]. The approach involves designing instructional activities aligned with these objectives, refining course content accordingly, and implementing assessment mechanisms to evaluate the achievement of these objectives. Such a structured framework strengthens teaching effectiveness, clarifies the roles of instructional personnel, and reinforces learning outcomes for students. As a result, students develop a clearer understanding of their learning process and are better equipped to apply their knowledge to meet individual needs [7, 11–16].

The ABO-OGS framework aligns seamlessly with GBL principles, as it provides specific, measurable objectives that guide both instruction and assessment in orthodontic education. The ABO-OGS, consisting of eight parameters, is widely recognized as a reliable method for assessing orthodontic treatment outcomes [8, 9, 17]. The accuracy of bracket positioning plays a crucial role in final treatment results, particularly concerning tooth alignment, marginal ridge height, and root parallelism [9, 17]. Errors in bracket positioning can lead to significant deviations in these three indicators. Therefore, the ABO-OGS evaluation system represents an ideal objective scoring framework for assessing the quality of orthodontic bracket bonding. However, the ABO-OGS-oriented bracket positioning teaching method remains underutilized in orthodontic education, particularly in the Chinese context.

In this study, we employed simplified ABO-OGS criteria (i.e., tooth alignment, marginal ridge height, and root parallelism) as guiding teaching objectives in orthodontic education. By aligning these criteria with GBL principles, we designed a course on orthodontic bracket bonding to evaluate the efficacy of three methods: traditional plaster model bonding, digital online bonding, and typodont bonding. The objective was to identify effective teaching methodologies and evaluation systems for bracket bonding, ultimately enhancing students’ practical skills and accelerating their mastery of orthodontic bracket bonding techniques. This endeavor aims to advance the overall quality of orthodontic education.

## Methods

### Research design

The laboratory course on orthodontic bracket bonding was conducted by 30 faculty members from the Orthodontics Teaching and Research Department at the School of Stomatology, Fujian Medical University. To ensure consistency and coherence in instruction, all educators participated in comprehensive centralized training. This training included collective lesson planning sessions, addressing standards for bracket positioning [18], procedure for bracket bonding, introduction of simplified ABO-OGS, and the grading criteria for laboratory course.

**Fig. 1:**
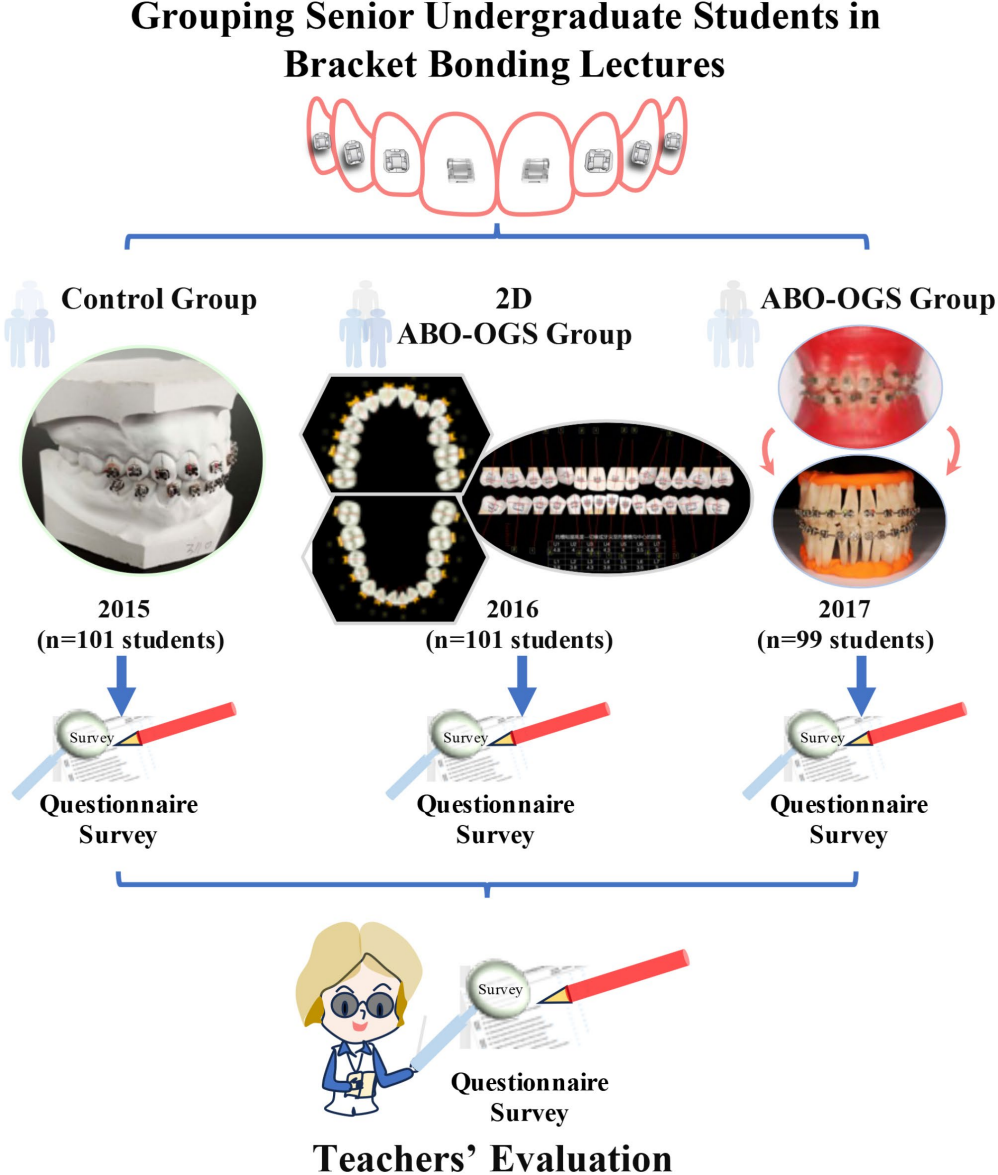
Schematic diagram depicting experimental design

The bracket bonding course targeted fourth-year undergraduate dental students from the academic years 2015 (101 students), 2016 (101 students), and 2017 (99 students). Teaching methodologies varied across cohorts (Fig. 1):

The 2015 cohort (Control Group) was instructed using traditional plaster models.

The 2016 cohort (Digital 2D ABO-OGS Group) received a digitally delivered course during the COVID-19 pandemic, due to restrictions on physical attendance.

The 2017 cohort (ABO-OGS Group) practiced on artificial teeth embedded in typodont wax, simulating clinical malocclusion.

Upon course completion, both students and instructors evaluated the effectiveness of the bracket bonding course using a standardized questionnaire (Supplementary Table 1) (Fig. 1). This evaluation assessed the outcomes of the different instructional methods and compared them across the three cohorts.

### Bracket bonding instruction methods

#### Traditional bracket bonding with plaster models

Students in the Control Group used standard plaster models with normal occlusion as their training tools. The clinical crown long axis of each tooth was marked with a pencil, and adhesive wax was applied to the base of the bracket for temporary bonding. After positioning, brackets (MBT prescription, 0.022 × 0.028-inch slot, Xinya Company, Hangzhou, China) were pressed to fit the tooth surface, and excess adhesive wax was removed (Supplemental Fig. 1). Instructors categorized performance into four grades (excellent, good, moderate, poor) based on clinical expertise and provided detailed score breakdowns within each category.

#### Simplified ABO-OGS-oriented bracket bonding in digital format

The digital 2D ABO-OGS Group utilized PowerPoint (PPT) 2016 software (Microsoft)^®^, USA for virtual simulations. The occlusal setup of teeth according to standard arch form and the buccal setup of teeth based on root parallelism with standard bracket position were established in PPT. Red crosses marked the standard bonding positions for each bracket, serving as references for correct bracket placement (Supplemental Fig. 2). Students placed brackets on teeth in simulated malocclusion and the positioning quality was evaluated according to simplified ABO-OGS.

#### Simplified ABO-OGS-oriented bracket bonding on typodont

The ABO-OGS Group worked on resin artificial teeth mounted on typodont wax models (Weierde, Fuzhou, China) simulating Angle’s Class I malocclusion (Supplemental Fig. 3A). The clinical crown long axis was marked on each tooth, and brackets were bonded using light-curing resin adhesive (Xihu, Hangzhou, China) (Supplemental Fig. 3B). Bracket positioning quality was evaluated by affixing a 0.0215 × 0.025-inch standard labial arch (Aimengdi, Shanghai, China) to the brackets, stabilizing the roots with playdough, and visually assessing according to simplified ABO-OGS.

### Simplified ABO-OGS for evaluation of bracket positioning

Bracket bonding quality was evaluated using the simplified ABO-OGS criteria, which included three components: tooth alignment, marginal ridge height, and root parallelism (Supplemental Fig. 4).

### Assessment of teaching efficacy

The efficacy of teaching methods was assessed through a “Teaching Satisfaction Questionnaire for Orthodontic Bracket Bonding Course” (Supplementary Table 1), comprising 18 items. The questionnaire evaluated teaching objectives, methods, efficacy, and overall satisfaction. A 6-point Likert scale (1 = strongly disagree to 6 = strongly agree) was used to record responses. Students and instructors participated voluntarily, and informed consent was obtained.

### Statistical analysis

Statistical analyses were conducted using SPSS 25 (Chicago, IL, USA), with statistical significance set at *p* < 0.05. Data were expressed as mean ± standard error. Descriptive statistics, including means and standard deviations for continuous variables and frequencies for categorical variables, were used to summarize key findings and enhance accessibility. Reliability of questionnaire responses was evaluated using the Intraclass Correlation Coefficient (ICC). Sample sizes were estimated and analyzed using G*Power (Heinrich-Heine-Universität Düsseldorf, Düsseldorf, Germany) based on pre-experimental results, with a statistical power of 0.8 and a significance level of 0.05.

The Shapiro-Wilk test was employed to assess data normality, while Levene’s test was used to evaluate the homogeneity of variances, which are prerequisites for parametric analyses. For data meeting these assumptions, a one-way ANOVA was performed to compare means across multiple groups. Post hoc pairwise comparisons were conducted using the Least Significant Difference (LSD) test to identify specific group differences. For non-parametric data that violated these assumptions, the Kruskal-Wallis test was utilized to compare medians between groups.

## Results

### Reliability and demographic characteristics of participants and questionnaires

The reliability of the data was evaluated using the Intraclass Correlation Coefficient (ICC), which was calculated for the scores of a randomly selected subset of ten participants, measured both before and after the experiment. The ICC values ranged from 0.511 to 1, with a mean ICC of 0.7793 ± 0.1393, demonstrating a high degree of reliability.

The demographic and academic characteristics of the three student cohorts are summarized in Supplemental Table 2. Statistical analysis indicated no significant differences in age, gender distribution, or pre-laboratory course performance among the cohorts (*p* > 0.05), confirming the comparability of the groups in terms of baseline characteristics.

Regarding the distribution of questionnaires, the control group (Class of 2015) yielded 83 valid responses. The experimental groups, consisting of the Classes of 2016 and 2017, were further subdivided. The 2D ABO-OGS subgroup of the Class of 2016 received 87 valid responses, while the ABO-OGS subgroup from the Class of 2017 collected 96 valid responses. For the teachers, a total of 30 questionnaires were distributed, and all were returned with valid responses.

### Limitations of digital 2D ABO-OGS bracket bonding in achieving pedagogical goals

**Fig. 2:**
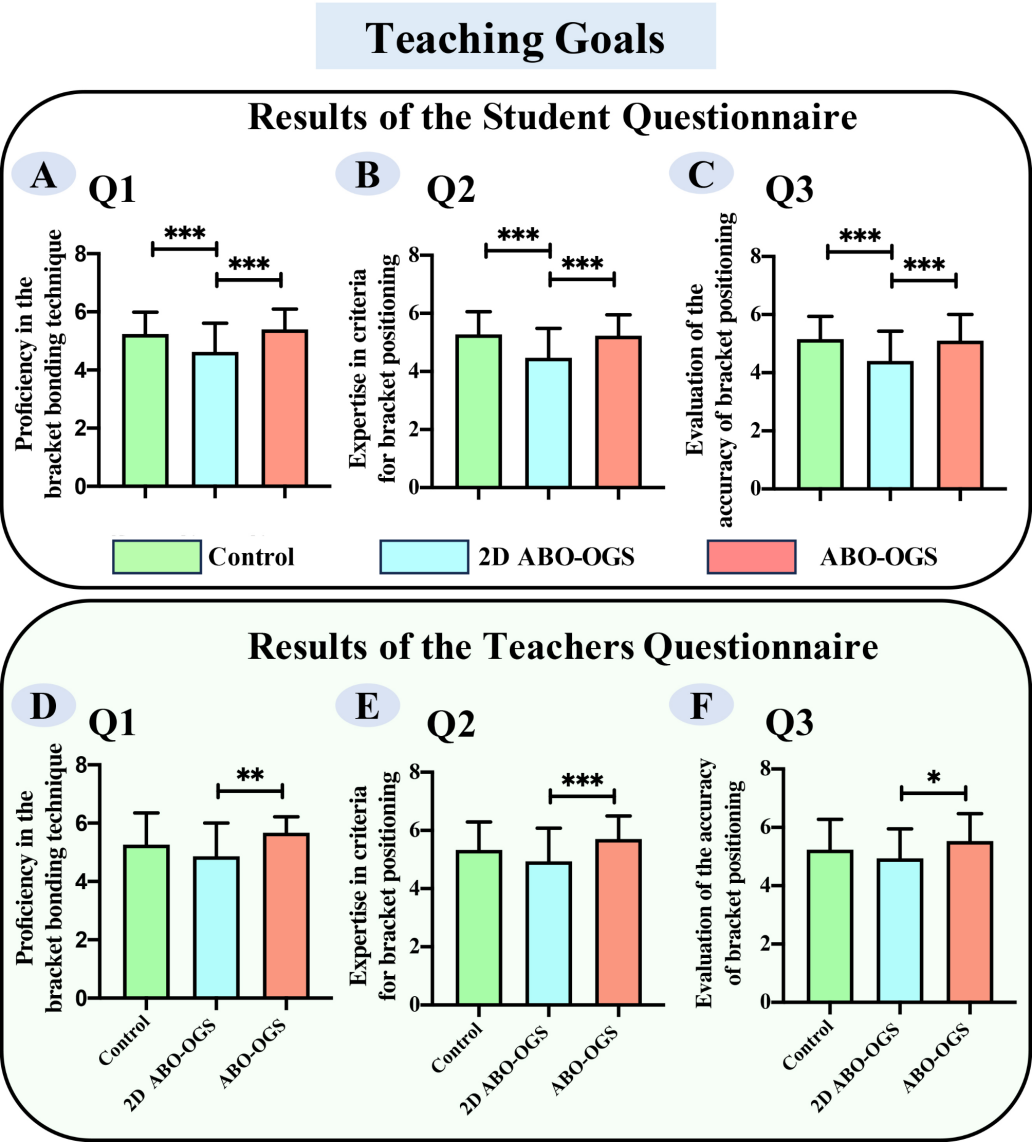
Statistical analysis of students’ and teachers’ feedback on teaching goals in the bracket bonding laboratory session. (Data were expressed as mean ± standard error. *p < 0.05, **p < 0.01, ***p < 0.001)

The majority of both students and teachers agreed that all three methods of bracket bonding effectively met the teaching objectives (Supplemental Fig. 5A and 6A). Fewer than 10% strongly disagreed or disagreed with the assertion that these experimental methods hindered students’ understanding of the bracket bonding process, the criteria for bracket positioning, or the ability to assess the accuracy of bracket placement (Supplemental Fig. 5A and 6A). However, further analysis revealed that both students and teachers perceived digital 2D ABO-OGS bracket bonding as less effective in achieving the teaching objectives when compared to the traditional method and the simplified ABO-OGS method (Fig. 2) (*p* < 0.05).

### Simplified ABO-OGS oriented method enhanced innovation and engagement in bracket bonding course

**Fig. 3:**
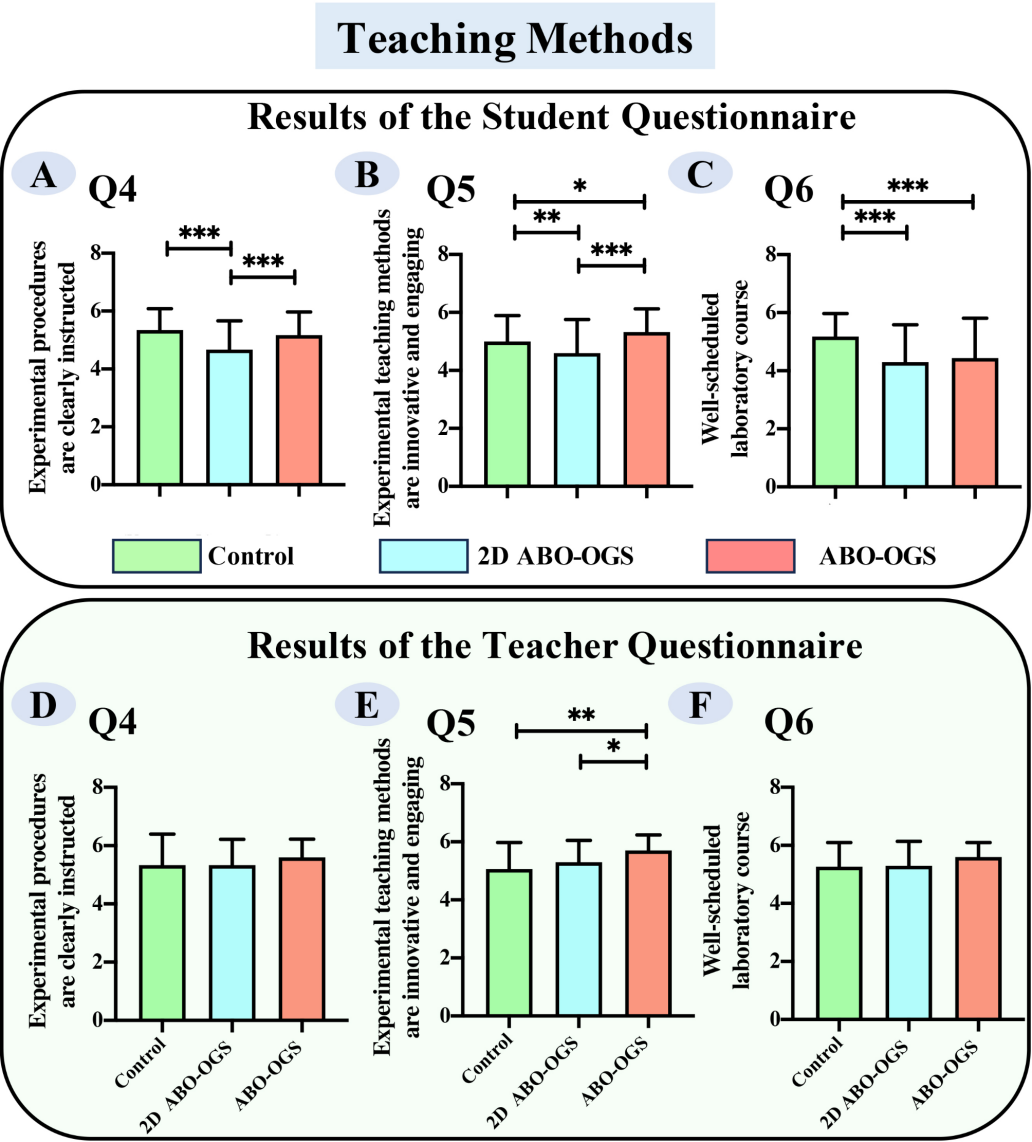
Statistical analysis of students’ and teachers’ feedback on teaching methods in the bracket bonding laboratory session. (Data were expressed as mean ± standard error. *p < 0.05, **p < 0.01, ***p < 0.001)

We assessed three key aspects of the teaching methodology: clarity of the experimental procedure explanation, novelty and engagement of the experimental teaching approach, and the appropriateness of the laboratory session schedule (Supplemental Fig. 5B and 6B, Fig. 3). This evaluation was conducted through a questionnaire, which revealed that both students and teachers rated the ABO-OGS group as superior to the traditional method and the 2D ABO-OGS methods in terms of novelty and engagement (Fig. 3B and E) (*p* < *0.05*). However, it is important to note that students perceived the ABO-OGS method as less optimized in terms of laboratory session scheduling, reporting that it required more time than the other methods (Fig. 3C) (*p* < *0.05*).

### ABO-OGS Group presented the highest effectiveness among three groups

**Fig. 4:**
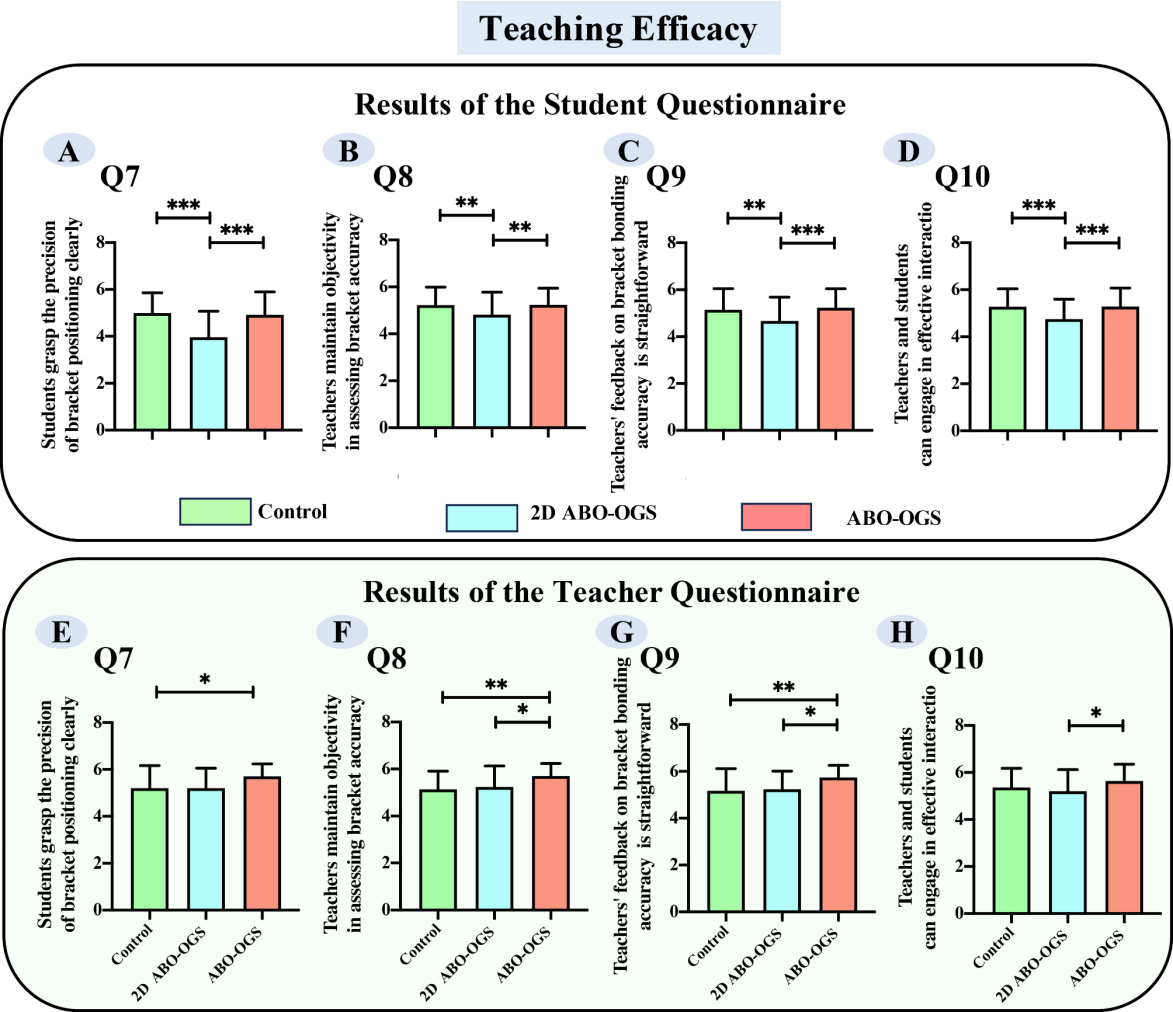
Statistical analysis of students’ and teachers’ feedback on teaching efficacy in the bracket bonding laboratory session. (Data were expressed as mean ± standard error. *p < 0.05, **p < 0.01, ***p < 0.001)

In terms of teaching efficacy, both students and teachers agreed that the digital 2D ABO-OGS method was less effective than the traditional method and ABO-OGS method in facilitating teacher-student interaction (Supplemental Fig. 5C and 6C, Fig. 4). Interestingly, there were differing perspectives on the statement, “Students are clear about the accuracy of their bracket positioning” (Fig. 4A and E). From the students’ standpoint, the digital 2D ABO-OGS method provided the least clarity (Fig. 4A), whereas teachers considered the traditional method to be the least effective (Fig. 4D) (*p* < 0.05). Additionally, differences were observed regarding the objectivity of teachers’ assessments (Fig. 4B and F). Students perceived the 2D ABO-OGS method as inferior to the other two methods in clarifying the criteria for bracket positioning accuracy (Fig. 4B) (*p* < 0.05). In contrast, teachers rated the traditional plaster model bonding technique as the least efficient, assigning it the lowest scores in this regard (Fig. 4F) (*p* < 0.05). Notably, an analysis of the combined responses from students and teachers to Q7 through Q10 indicates that the ABO-OGS teaching method is more effective than the other two approaches.

### ABO-OGS method enhanced students’ handling skills and improved satisfaction among students and instructors

**Fig. 5:**
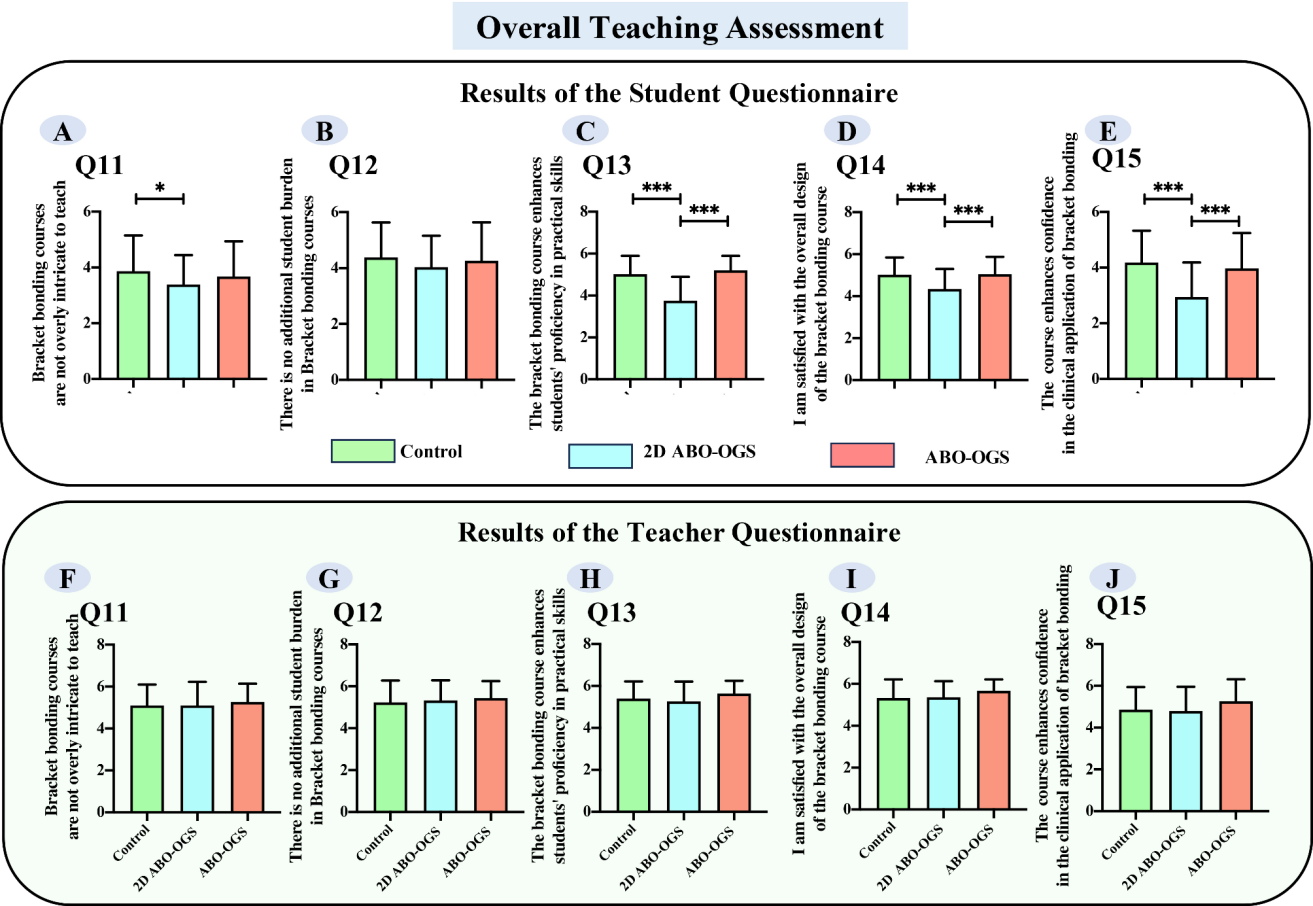
Statistical analysis of students’ and teachers’ feedback on overall teaching assessment in the bracket bonding laboratory session. (Data were expressed as mean ± standard error. *p < 0.05, **p < 0.01, ***p < 0.001)

The results of the overall teaching assessment showed that most students and instructors believed the ABO-OGS bracket bonding technique effectively enhanced practical skills and were generally satisfied with its implementation (Supplemental Fig. 5D and 6D). However, students found the 2D ABO-OGS method to be overly complex and burdensome, which they felt hindering the development of their practical skills and future career prospects (Fig. 5A) (*p* < 0.05). In contrast, teachers reported that ABO-OGS group achieved higher scores compared to the other two groups, although with no significant differences among the three experimental groups (Fig. 5F and J) (*p* > 0.05).

### Simplified ABO-OGS satisfies teachers and students high as evaluation criteria

**Fig. 6:**
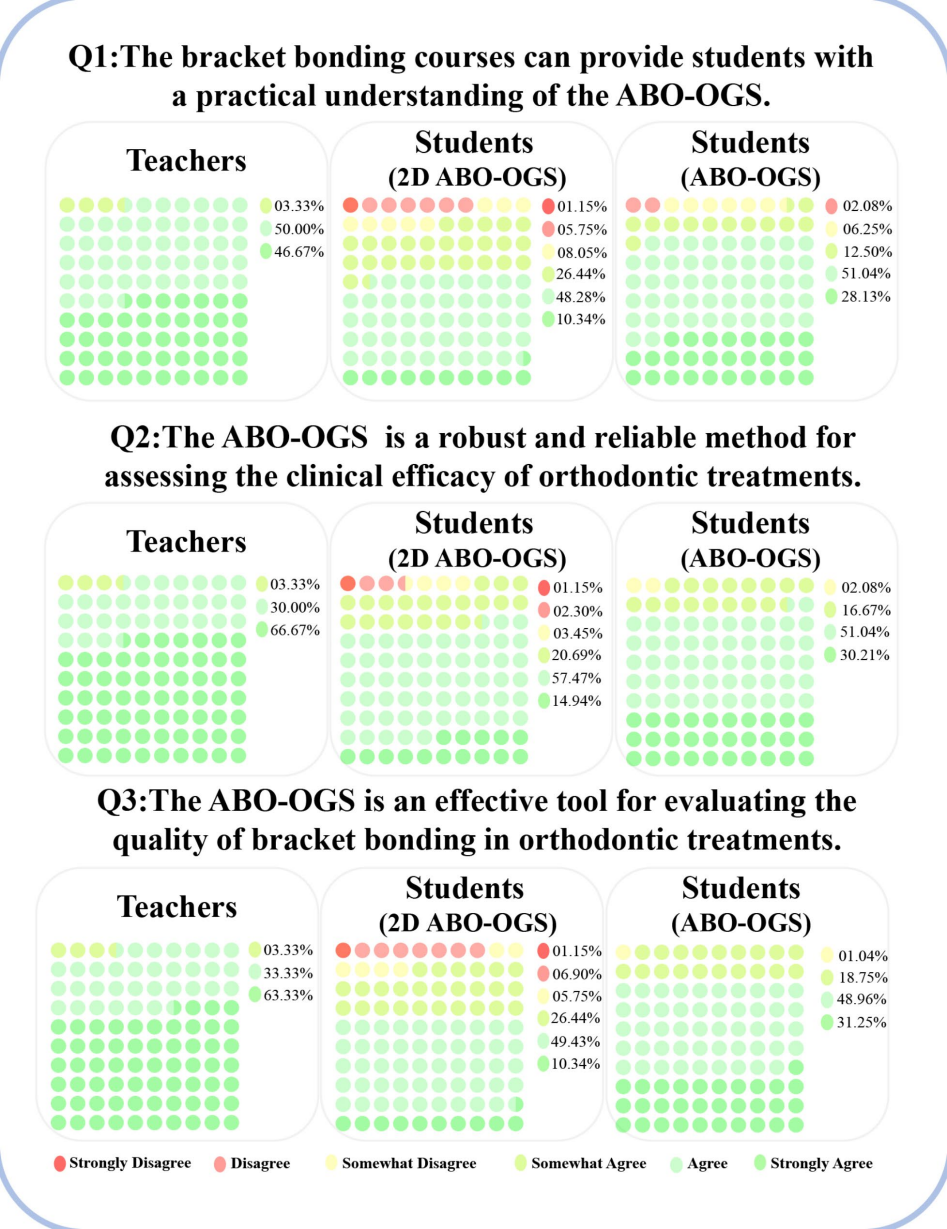
Percentage of student and teacher satisfaction with simplified ABO-OGS standard

**Fig. 7:**
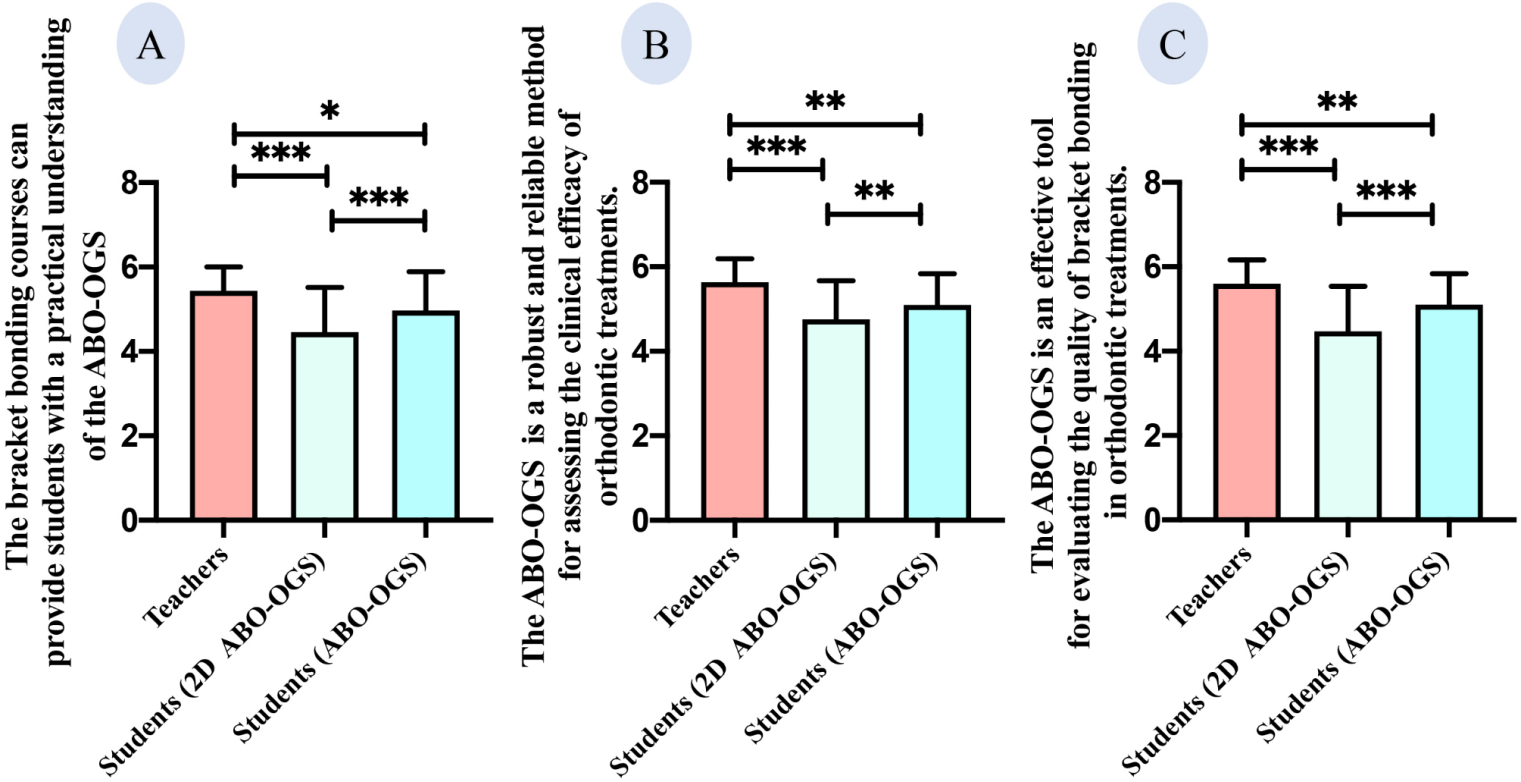
Comparative analysis of student and teacher feedback on simplified ABO-OGS standards. (Data were expressed as mean ± standard error. *p < 0.05, **p < 0.01, ***p < 0.001)

Both students and instructors recognize the simplified ABO-OGS as a critical tool for evaluating orthodontic clinical outcomes and bracket bonding (Fig. 6). Bracket bonding courses are instrumental in providing a thorough understanding and mastery of the ABO-OGS evaluation criteria. Notably, students who participated in online bracket bonding lab sessions due to the pandemic rated their understanding of the ABO-OGS lower than those who attended in-person sessions, as well as their instructors (Fig. 7) (*p* < 0.05).

## Discussion

Orthodontic bracket bonding is critical for achieving optimal results in orthodontic straight arch techniques. Mastery of this technique serves as a gateway for dental students to enter orthodontic clinical practice, making the orthodontic bracket bonding laboratory course a cornerstone of their education [1–4]. While the traditional plaster bracket bonding technique is economical and convenient for teaching, its effectiveness heavily relies on the clinical experience of the instructor [5, 6, 14]. As a result, students often lack clear, objective feedback on the correctness of their bracket positioning, especially regarding tooth root direction after bonding.

For undergraduate dental students, the major teaching objective is to help them get acquainted with the procedure and realize the importance of precise bracket positioning. To address these challenges, we employed the simplified ABO-OGS, a widely accepted index for achieving high standards in orthodontic treatment outcomes, as the benchmark for bracket bonding in this study. While the wax typodont articulator is widely used for preclinical hands-on training in orthodontics, its implementation is costly and time-consuming for undergraduates due to limited resources and class time. Consequently, we opted not to use the full typodont set in this study. Instead, we compared the effects of three different bracket bonding experimental sessions: traditional plaster model bonding (Control Group), online 2D digital bracket bonding (2D ABO-OGS Group), and artificial tooth bonding (ABO-OGS Group). Our findings indicate that all three types of experimental sessions effectively achieved the teaching objectives and produced satisfactory results.

Objective-oriented teaching facilitates effective educational outcomes [14–16]. The Objective Grading System (OGS), proposed by the American Board of Orthodontics in 1994, is now considered a standard tool for evaluating orthodontic treatment results [8, 9]. This study aimed to simplify the ABO-OGS by focusing on tooth alignment, marginal ridge heights, and root parallelism in orthodontic bracket bonding. Both teachers and students highly rated this methodology, with students in the ABO-OGS group appreciating the immediate visual feedback on how their bracket positioning influenced tooth root movement after full engagement and expression of bracket presetting. This approach incorporating similar procedure as clinic operation and objective parameterized evaluation system was perceived as an engaging and effective laboratory session that enhanced their technical proficiency.

Proper bracket positioning is crucial for effective orthodontic treatment. In orthodontics, indirect bonding technique offers numerous advantages over direct bonding, such as chair time saving, a more precise bracket placement and removal of flash to the bracket bases [19]. Additionally, the two techniques showed similar bonding failures [20]. Technological advancements in orthodontics, such as 3D digital indirect bonding, offer higher accuracy in bracket placement and are becoming increasingly popular [21–25]. However, due to time and financial constraints, this technique was not employed in our study. We recommend incorporating 3D digital methods into future laboratory sessions to improve the quality of orthodontic education. Besides, although promising, the scalability and cost-effectiveness of these 3D techniques remain a critical consideration for widespread adoption in educational settings. Further studies are needed to evaluate the practical challenges of implementing these technologies in resource-limited environments.

Particularly, our study found that both teachers and students provided less favorable feedback on the digital 2D bracket bonding sessions compared to traditional plaster model bonding and artificial tooth bonding with standard arch-wire verification. A significant factor influencing this feedback may be the abrupt shift to online classes during the pandemic, which hindered students’ ability to quickly adapt to and effectively master the online bracket bonding technique [26–28]. This underscores the hands-on nature of orthodontic training, where students can only achieve proficiency through direct practice.

Previous studies have highlighted several limitations of online teaching in medical and dental education, including the lack of immediate feedback and personalized guidance that in-person instruction offers [29–32]. The tactile and visual aspects of orthodontic procedures are challenging to convey through digital platforms, often leading to misunderstandings or improper technique application. Furthermore, the absence of physical models and realistic clinical simulations limits the online learning experience [33, 34]. Technical issues, such as internet connectivity problems, varying levels of access to necessary equipment, and the lack of a controlled learning environment, further hinder the effectiveness of online training [31, 33, 34]. As a result, students may feel isolated and disengaged, diminishing the overall learning experience.

Despite these limitations, digital learning offers notable advantages, such as convenience, repeatability and time efficiency [28, 35]. However, it is insufficient for developing the practical skills required in orthodontic training. One study demonstrated that blended online-offline teaching in medicinal chemistry significantly improved academic performance and student satisfaction [36]. Similarly, blended teaching in clinical skill training has been shown to achieve positive pedagogical outcomes in medical education [37–39]. Based on our findings, we recommend the integration of online and offline methodologies within the framework of simplified ABO-OGS-oriented bracket bonding lectures. Precisely, prior to their participation in the ABO-OGS laboratory course, students should be encouraged to engage with and familiarize themselves with the digital 2D ABO-OGS laboratory course materials at home. This preparatory step will serve to facilitate a more profound comprehension of the simplified ABO-OGS bracket bonding procedures, ultimately enhancing the overall educational experience. This blended learning approach may improve overall learning efficacy by providing students with fundamental theoretical knowledge through digital platforms, while concurrently reinforcing practical skills through hands-on training sessions.

Finally, while our study emphasizes the importance of hands-on practice, we must acknowledge its limitations. The study’s small sample size and the constraints imposed by the pandemic, such as the rapid shift to online learning, may have influenced the outcomes. Future research with larger sample sizes and more diverse instructional settings would provide more robust data on the effectiveness of different teaching methods. The limited time of the course and the confined objective for undergraduate students also hindered the full application of ABO-OGS in evaluation of orthodontic bonding outcomes. Further improvement of the ABO-OGS oriented orthodontic bracket bonding course in digital 3D simulation software and in full typodont training of orthodontic postgraduate and residency students is the direction of our future efforts.

## Conclusions

Both traditional and simplified ABO-OGS oriented courses on bracket bonding yielded favorable results, with the simplified ABO-OGS approach being more engaging and innovative. The findings emphasize the critical role of hands-on practice in achieving optimal proficiency in bracket bonding techniques. Incorporation advanced methods, such as 3D digital brackets bonding, into future lab sessions will enhance the learning process. Hybrid educational model that integrates 2D and 3D digital ABO-OGS oriented training with offline practical sessions shows considerable potential for qualified training and examination for orthodontic residency students.

## Electronic supplementary material

Below is the link to the electronic supplementary material.

Supplementary Material 1

## Data Availability

Data generated or analyzed during this study can be obtained from the corresponding author upon reasonable request.

## Abbreviations

ABO-OGS: American Board of Orthodontics Objective Grading System
GBL: Goal-Based Learning
ICC: Intraclass Correlation Coefficient
LSD: Least Significant Difference
PPT: PowerPoint
