# Supplementary material for: Simplified ABO-OGS orientation improves training of orthodontic bracket positioning for undergraduate dental students
Source: BMC Med Educ. 2025 Feb 20;25:280. doi: 10.1186/s12909-025-06839-y (PMC11844120; doi:10.1186/s12909-025-06839-y)
Supplement: Supplementary file 1 — Supplementary Material 1 [file 12909_2025_6839_MOESM1_ESM.pdf]

Supplemental Materials

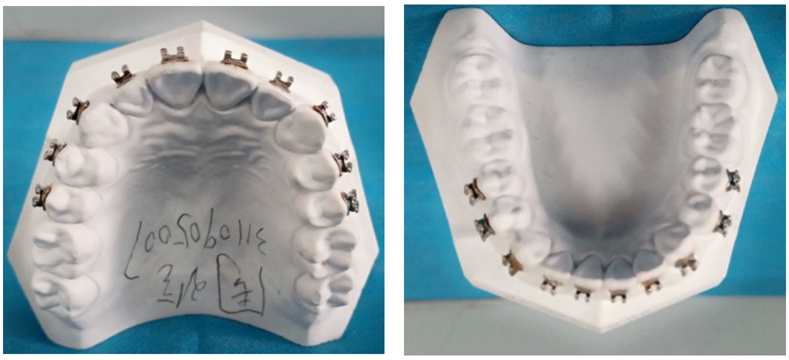

Supplemental Figure 1: Schematic Representation of Traditional Plaster Model Bracket Bonding Teaching

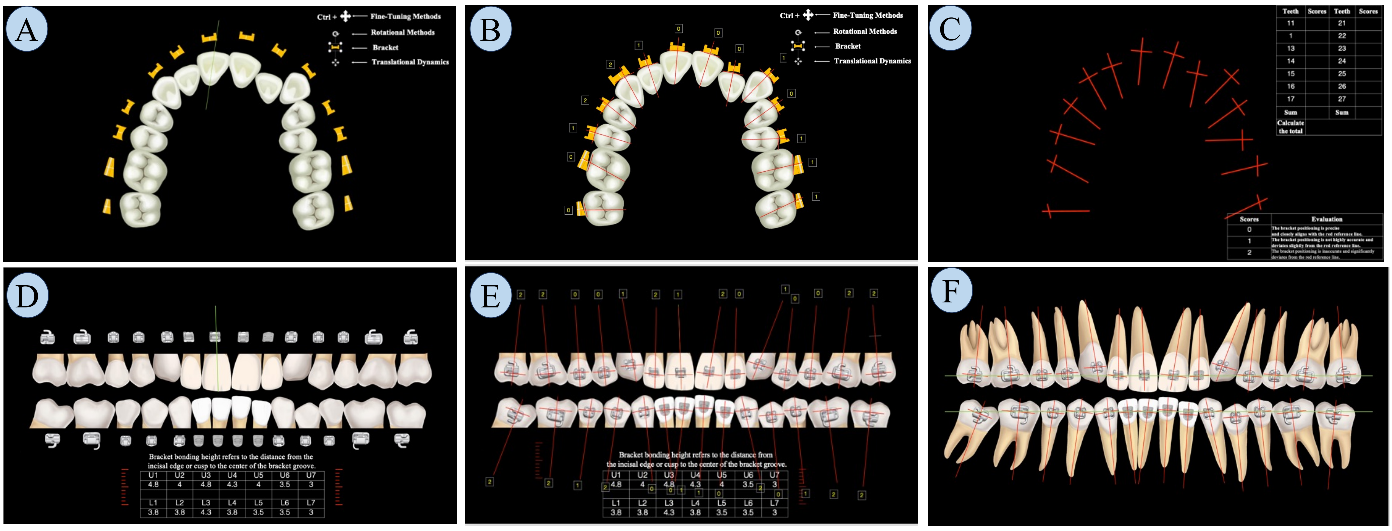

Supplemental Figure 2: Schematic of Simplified ABO-OGS Online Bracket Bonding Procedure. A/D: Pre-bracket bonding; B/E: Student online bracket bonding assignment; C/F: Standardized answer for bracket bonding.

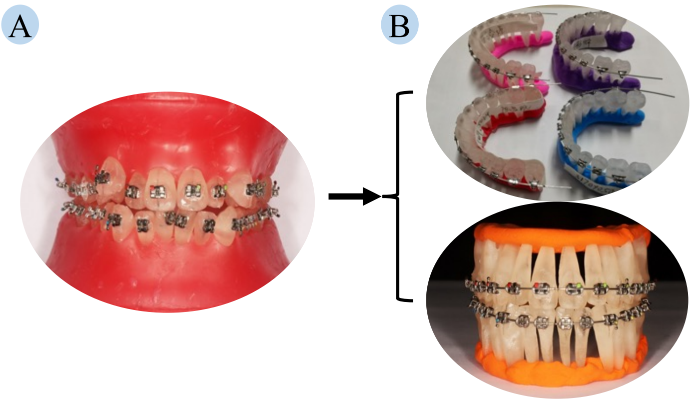

Supplemental Figure 3: Simplified ABO-OGS-Oriented Bracket Bonding on Artificial Teeth Utilizing Standard Labial Arch: A) Bracket bonding to Typdont model displaying Angle's Class 1 malocclusion. B) Coupling of artificial teeth with bonded brackets using a standard labial arch to assess tooth alignment, root parallelism, and employing rubber cementation at tooth roots for enhanced anchorage.

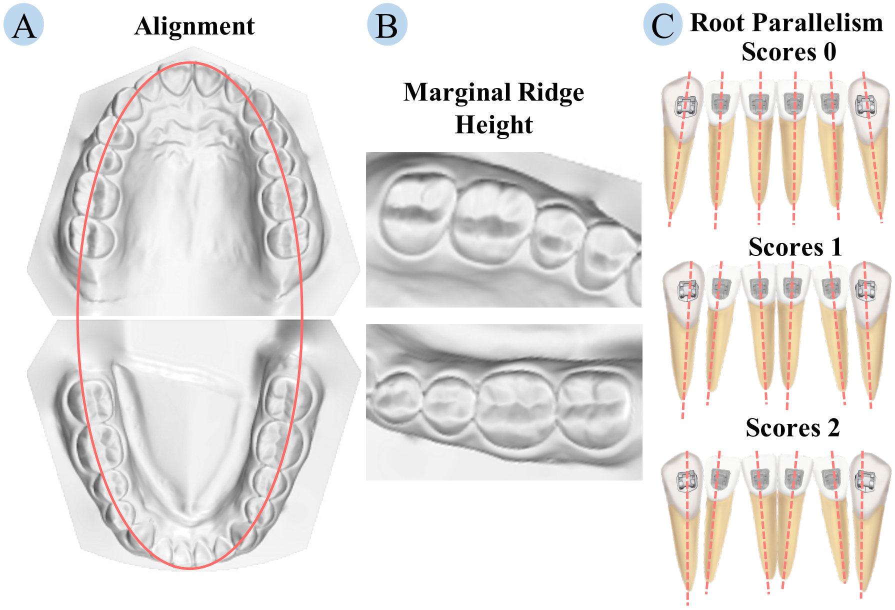

Supplemental Figure 4: Schematic of Simplified ABO-OGS: A) Optimal tooth alignment. B) Adequate marginal ridge height. C) Deduction of root parallelism.

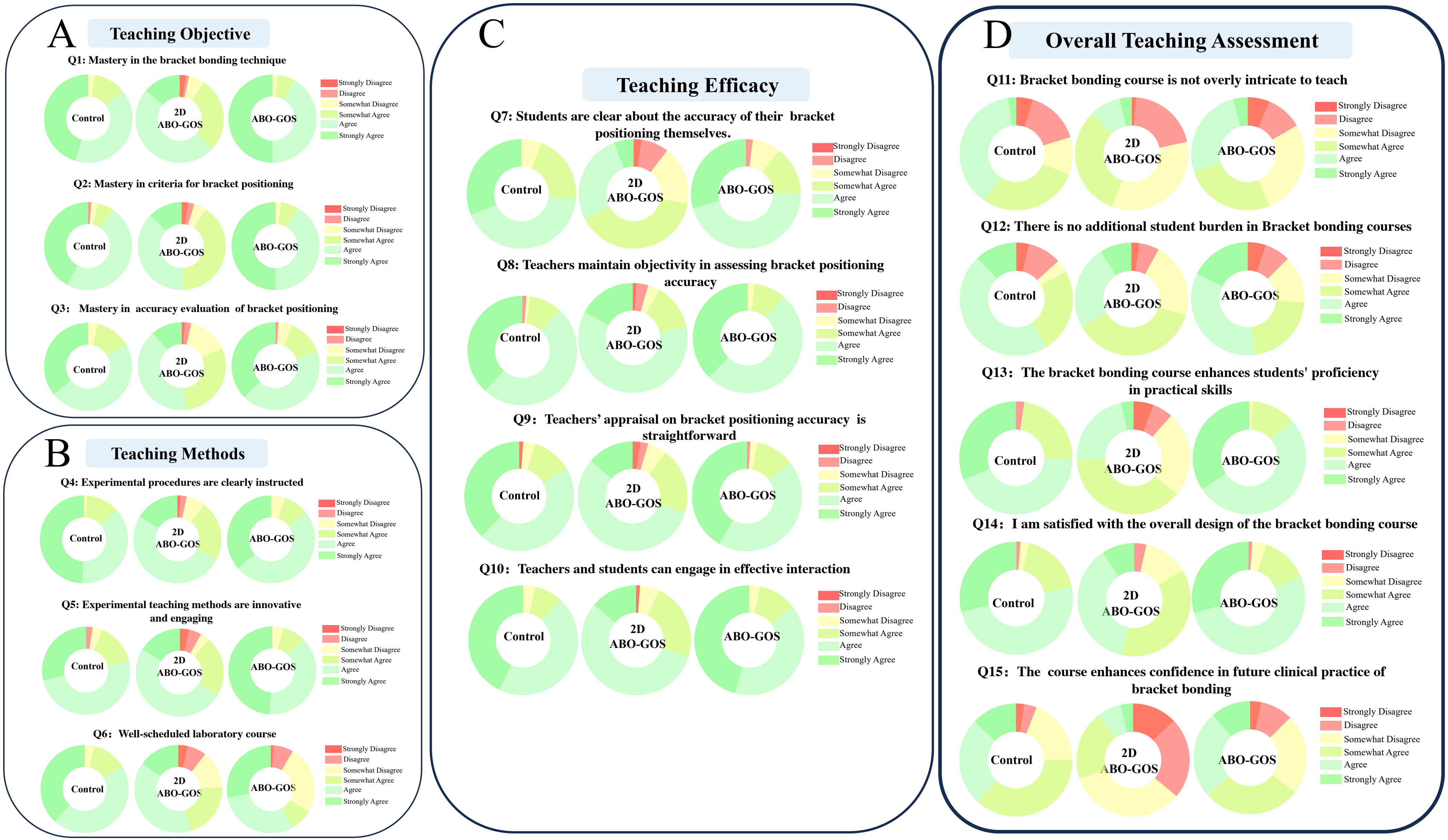

Supplemental Figure 5: Percentage of Students' Satisfaction with the Effectiveness of Their Respective Bracket Bonding Lectures

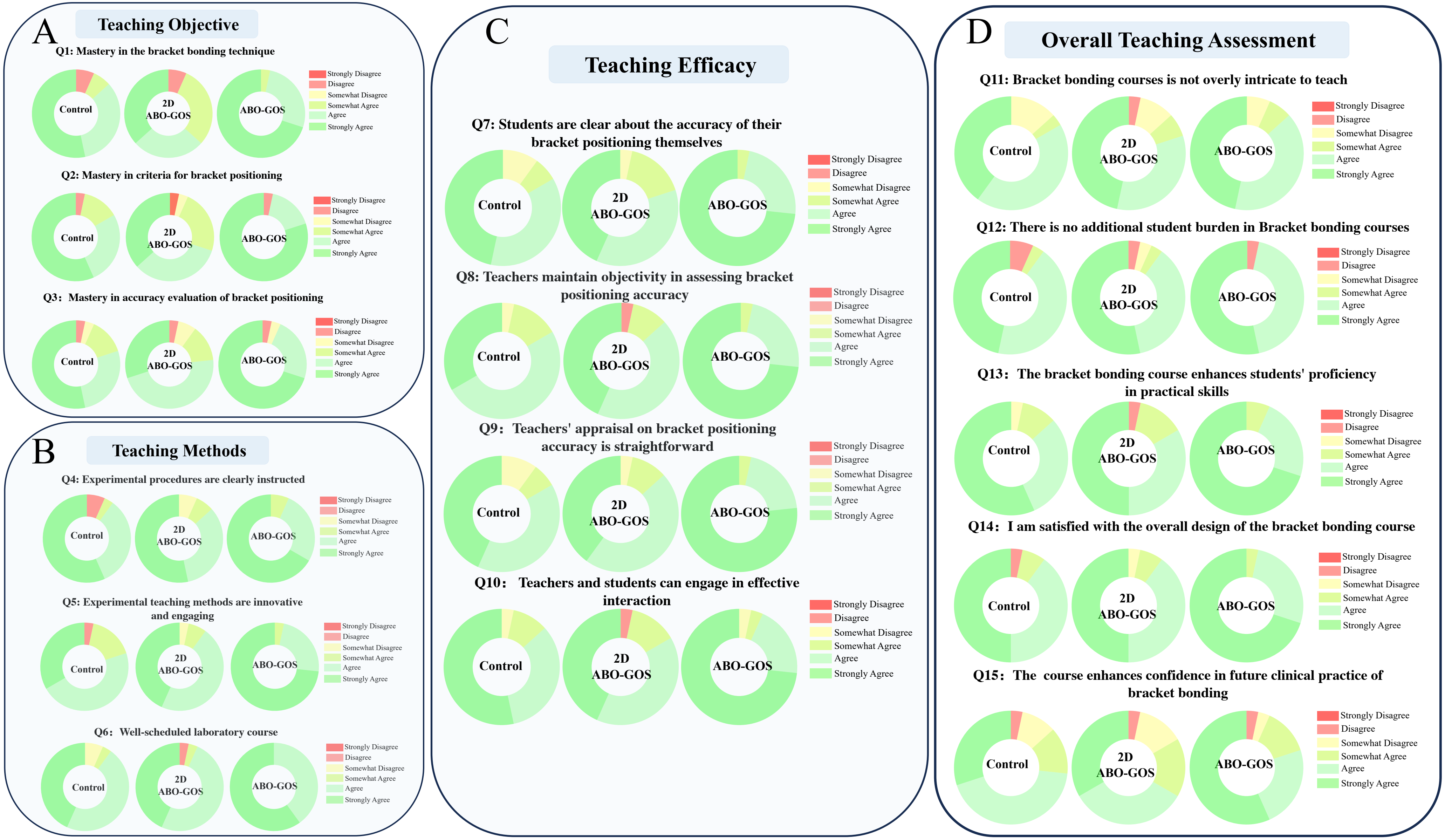

Supplemental Figure 6: Percentage Analysis of Teachers' Satisfaction with Teaching Effectiveness in the Lectures on Three Types of Bracket Bonding.

Supplemental Table1: Satisfaction Survey on Teaching in Orthodontic Bracket Bonding Laboratory Course

	Evaluation item	Strongly Disagreed	Disagreed	Somewhat Disagreed	Somewhat Agreed	Agreed	Strongly Agreed
Teaching Objective	Q1: Mastery in bracket bonding technique.						
	Q2: Mastery in criteria for bracket positioning.						
	Q3: Mastery in accuracy evaluation of bracket positioning.						
Teaching Methods	Q4: Experimental procedures are clearly instructed.						
	Q5: Experimental teaching methods are innovative and engaging.						
	Q6: Well-scheduled laboratory course.						
Teaching Efficacy	Q7: Students are clear about the accuracy of their bracket positioning themselves.						
	Q8: Teachers maintain objectivity in assessing bracket positioning accuracy.						
	Q9: Teachers' appraisal on bracket positioning accuracy is straightforward						
	Q10: Teachers and students can engage in effective interaction.						
Overall Teaching Assessment	Q11: Bracket bonding course is not overly intricate.						
	Q12: There is no additional student burden in Bracket bonding course.						
	Q13: The bracket bonding course enhances students' proficiency in practical skills.						
	Q14: I am satisfied with the overall design of the bracket bonding course.						
	Q15: The course enhances confidence in future clinical practice of bracket bonding.						

Supplemental Table 2: Basic Information of Participants in different methods of bracket boding

	Control Group		Experimental Group			
			2D ABO-OGS		ABO-OGS	
Age (yr)	22.75±1.46[a]		22.69±1.75[a]		22.80±1.47[a]	
Previous Scores	80.34±3.45[b]		81.764±4.32[b]		80.94±2.12[b]	
Gender Distribution (%)	43.56[c] (male)	56.43[C] (female)	38.61[c] (male)	61.39[C] (female)	33.33[c] (male)	66.67[C] (female)

(Identical letters within the same row indicate no statistically significant differences in pairwise comparisons.)
